# Comparison of Compartmental and Non-Compartmental Analysis to Detect Biopharmaceutical Similarity of Intravenous Nanomaterial-Based Rifabutin Formulations

**DOI:** 10.3390/pharmaceutics15041258

**Published:** 2023-04-17

**Authors:** Nadezhda Osipova, Andrey Budko, Olga Maksimenko, Elena Shipulo, Ludmila Vanchugova, Wenqian Chen, Svetlana Gelperina, Matthias G. Wacker

**Affiliations:** 1 Nanosystem Ltd., Kolomenskiy Proezd 13A, 115446 Moscow, Russia; kompacc@yandex.ru (N.O.); omnews@mail.ru (O.M.); elenashipulo@rambler.ru (E.S.); vanchugowa.lyudmila@yandex.ru (L.V.); 2 N.N. Blokhin Russian Cancer Research Center, Russian Academy of Medical Science, Kashirskoye Shosse 24, 115478 Moscow, Russia; apbudko@mail.ru; 3 Department of Pharmacy, Faculty of Science, 4 Science Drive 2, Singapore 117544, Singapore; wen.chen@nus.edu.sg

**Keywords:** nanoparticles, rifabutin, population modeling, modeling, bioequivalence, injectables, safety, biodistribution

## Abstract

Pharmacometric analysis is often used to quantify the differences and similarities between formulation prototypes. In the regulatory framework, it plays a significant role in the evaluation of bioequivalence. While non-compartmental analysis provides an unbiased data evaluation, mechanistic compartmental models such as the physiologically-based nanocarrier biopharmaceutics model promise improved sensitivity and resolution for the underlying causes of inequivalence. In the present investigation, both techniques were applied to two nanomaterial-based formulations for intravenous injection, namely, albumin-stabilized rifabutin nanoparticles and rifabutin-loaded PLGA nanoparticles. The antibiotic rifabutin holds great potential for the treatment of severe and acute infections of patients co-infected with human immunodeficiency virus and tuberculosis. The formulations differ significantly in their formulation and material attributes, resulting in an altered biodistribution pattern as confirmed in a biodistribution study in rats. The albumin-stabilized delivery system further undergoes a dose-dependent change in particle size which leads to a small yet significant change in the in vivo performance. A second analysis was conducted comparing the dose fraction-scaled pharmacokinetic profiles of three dose levels of albumin-stabilized rifabutin nanoparticles. The dose strength affects both the nanomaterial-related absorption and biodistribution of the carrier as well as the drug-related distribution and elimination parameters, increasing the background noise and difficulty of detecting inequivalence. Depending on the pharmacokinetic parameter (e.g., AUC, C_max_, Cl_obs_), the relative (percentage) difference from the average observed using non-compartmental modeling ranged from 85% to 5.2%. A change in the formulation type (PLGA nanoparticles vs. albumin-stabilized rifabutin nanoparticles) resulted in a similar level of inequivalence as compared to a change in the dose strength. A mechanistic compartmental analysis using the physiologically-based nanocarrier biopharmaceutics model led to an average difference of 152.46% between the two formulation prototypes. Albumin-stabilized rifabutin nanoparticles tested at different dose levels led to a 128.30% difference, potentially due to changes in particle size. A comparison of different dose strengths of PLGA nanoparticles, on average, led to a 3.87% difference. This study impressively illustrates the superior sensitivity of mechanistic compartmental analysis when dealing with nanomedicines.

## 1. Introduction

The antibiotic rifabutin (spiro-piperidyl-rifamycin) belongs to the rifamycin family and is mainly used for the treatment of acute *Mycobacterium tuberculosis* and disseminated *Mycobacterium avium complex* (MAC). Although its current applications are very limited, recent evidence indicates that in combination with other drugs, synergistic effects are to be expected [1]. Compared to rifampicin, rifabutin has a much lower bioavailability, improved cell penetration, and higher tissue distribution [2,3].

The added value of rifabutin arises from the reduced induction of CYP3A enzymes [4]. Hence, in contrast to rifampicin, rifabutin has little effect on serum concentrations of current protease inhibitors and provides a reasonable treatment option for patients co-infected with human immunodeficiency virus (HIV) and tuberculosis [1]. Among adults who require protease inhibitors as part of their HIV therapy, rifabutin is recommended by the World Health Organization for the prevention of disseminated MAC infection [5]. Moreover, as compared to rifampicin, rifabutin exhibits a higher activity against non-tuberculous mycobacteria, such as MAC or *Mycobacterium abscessus* (MAB). This higher activity is likely due to differences in mycobacterial cell pharmacokinetics. Rifampicin appears to be more readily metabolized by MAB than rifabutin [1,6,7].

In the context of the present investigation, we developed two different formulation prototypes for intravenous injection, namely, albumin-stabilized rifabutin nanoparticles and rifabutin-loaded PLGA nanoparticles. They exhibit considerable differences in their material and quality attributes. The albumin-stabilized delivery system was prepared using the nab technology [8] and undergoes a dose-dependent change in particle size, which results in a small yet significant change in the in vivo performance at different dose strengths. Both nanomedicines of the poorly soluble drug rifabutin [9] have great potential in the treatment of acute and severe infections. Not unlike many other non-oral delivery systems, such as liposomal amphotericin (AmbiSome^®^), paclitaxel encapsulated in albumin nanoparticles (Abraxane^®^), or PEG-PLGA micelles (Genexol-PM^®^), they are characterized by an altered biodistribution pattern that is difficult to detect from plasma pharmacokinetics alone. In preclinical and clinical research, plasma concentration-time profiles are commonly analyzed through non-compartmental analysis (NCA), which involves the determination of selected parameters, such as C_max_, t_max_, and AUC, to assess differences in their biopharmaceutical behavior. Despite the advantages associated with an unbiased and highly reproducible data evaluation [10], it should be noted that the sensitivity and robustness of NCA in detecting formulation-related differences among various nanomedicines are limited. Alternatively, compartmental pharmacokinetic models, such as the physiologically-based nanocarrier biopharmaceutics (PBNB) model [11,12,13,14], can be utilized. However, these models involve certain mechanistic assumptions regarding the distribution and elimination behavior of the dosage form in comparison to the free drug, which may introduce bias in data analysis. Nevertheless, this approach is likely to enhance sensitivity in detecting biopharmaceutical inequivalence while minimizing the impact of other confounding factors. Here, we compared the two nanoparticle formulations of rifabutin at various dose strengths using both NCA and mechanistic compartmental analysis. The sensitivities of both methods for the detection of biopharmaceutical inequivalence from plasma pharmacokinetics were evaluated in three settings. Firstly, we compared the two formulation types (PLGA nanoparticles and albumin-stabilized particles) which is a major inequivalence due to differences in composition and physicochemistry. Secondly, we used the dose fraction-scaled profiles to compare albumin-stabilized nanoparticles at different dose strengths. Our characterization data confirms that this may lead to a minor change in the particle size. Thirdly, we compared PLGA nanoparticles at three different dose strengths which change drug-related parameters without affecting the material or physicochemical characteristics of the delivery system.

## 2. Materials and Methods

### 2.1. Rifabutin-Loaded PLGA Nanoparticles

The rifabutin-loaded PLGA nanoparticles were produced by a high-pressure o/w emulsification/solvent evaporation technique. An amount of 1.5 g of PLGA polymer (Resomer^®^ 502H: D,L-lactide-co-glycolide, 50:50 mol/mol; molecular weight 7–17 kDa; η = 0.21 dL/g, Evonik Röhm GmbH, Darmstadt, Germany) and 0.6 g of rifabutin (rifabutin, Luohe Nanjiecun Pharmaceutical Group Pharmacy Co., Ltd., Luohe Henan, China) was dissolved in 30 mL of dichloromethane. The organic phase was poured into a 1% aqueous HSA solution (150 mL), and the mixture was emulsified using a high-shear rotor-stator mixer (UltraTurrax T18 Basic, IKA Industrie- und Kraftfahrzeugausrüstung GmbH, Königswinter, Germany) for 5 min at 23,000 rpm. Afterward, the emulsion was passed through a high-pressure homogenizer (Panda PLUS 2000, GEA Niro Soavi, Parma PR, Italy) at 1000 bar for 5 min. After evaporation of the organic solvent under vacuum (Rotavapor R-210/V, Buchi, Flawil, Switzerland), the suspension was filtered through a glass porous filter (pore size 90–150 µm) and 1% mannitol was added as a cryoprotectant. The suspension was freeze-dried for 48 h (Alpha 2-4 LSC, Martin Christ GmbH, Osterrode, Germany).

### 2.2. HSA-Stabilized Rifabutin Nanoparticles

The HSA-stabilized rifabutin nanoparticles were produced by nanoprecipitation. A concentrated solution of rifabutin in ethanol (80 mg⸱mL^−1^, 10.5 mL) was added to a 3% aqueous solution of HSA (125 mL). The HSA solution was obtained by dilution of a commercial product (20% HSA solution, Baxter, Wien, Austria). During and until 1 h after the addition of rifabutin, the solution was continuously agitated using a magnetic stirrer (550 rpm). The residual organic solvent was partially removed under a vacuum. The resulting suspension was filtered through a glass porous filter (pore size 90–150 µm) and freeze-dried as described above.

### 2.3. Characterization of Nanoparticle Formulations

The particle size and polydispersity index (PDI) were measured by dynamic light scattering (Zetasizer Nano ZS, Malvern Instruments, Malvern, UK) in MQ-water. The zeta potential was determined by microelectrophoresis using disposable cuvettes (U-shaped folded capillary zeta cells). All measurements were performed in quadruplicates without dilution or with a 50-fold dilution of the suspension.

### 2.4. Evaluation of Drug Content and Encapsulation Efficiency

The drug content of HSA-stabilized rifabutin nanoparticles was determined after the dissolution of freeze-dried nanoparticles in DMSO. Precipitated albumin was separated by centrifugation (centrifuge 5415R, Eppendorf, Hamburg, Germany; 13,200× *g* rpm, 18 °C, 20 min). The rifabutin concentration was measured by spectrophotometry (λ_max_ = 520 nm) using a calibration curve in the concentration range from 0.005 to 0.27 mg⸱mL^−1^ (A = 0.0038 × C, correlation coefficient R^2^ = 0.9998). To determine the concentration of free (unbound) rifabutin in the aqueous phase, 1 mL of a stable rifabutin nanoparticle suspension was centrifuged at 13,200× *g* rpm, 5 °C for 30 min followed by the quantification of the drug from the supernatant.

Rifabutin-loaded PLGA nanoparticles were resuspended in distilled water; 0.4 mL of the suspension was taken, placed in an ultrafiltration device (Microcon MWCO 30 kDa filters, Millipore, Burlington, MA, USA), and nanoparticles were separated by centrifugation (centrifuge 5415R, Eppendorf, Hamburg, Germany) at 13,200× *g* rpm, 25 °C for 30 min. The rifabutin concentration in the filtrates was measured spectrophotometrically at 513 nm using a calibration curve in the concentration range from 0.1 to 0.65 mg⸱mL^−1^ (A = 0.0036 × C, correlation coefficient R^2^ = 0.9980). The determination was repeated three times (*n* = 3). Drug encapsulation efficiency (EE, %) was calculated as follows:

(1) $$\mathrm{EE} [\%]=\frac{C_{\mathrm{total}}-{\;C}_{\mathrm{free}}}{C_{\mathrm{total}}}\times 100\%$$

where EE (%) denotes the encapsulation efficiency, C_total_ (mg⸱mL^−1^) is the total rifabutin content in the sample, C_free_ (mg⸱mL^−1^) is the concentration of free rifabutin.

### 2.5. In Vitro Drug Release Study

The in vitro release of rifabutin from the PLGA nanoparticles was evaluated in a mixture of 0.01 M phosphate buffer with 0.9% NaCl (PBS) and 0.1% ascorbic acid (pH = 7.4). The freeze-dried nanoparticles were resuspended in the release medium and further diluted to a rifabutin concentration of ≈154 µg/mL and a final volume of 25 mL, followed by incubation at +37 °C under continuous shaking. At predetermined time intervals (1, 2, 3, 4, 6, 24 h), the nanosuspension aliquots were sampled, and the nanoparticles were separated by centrifugation (20,000× *g* rpm/48,254× *g* for 30 min at +5 °C, Avanti J-30I, Beckman Coulter, Pasadena, CA, USA). The concentration of rifabutin in the supernatant was measured spectrophotometrically at λ = 513 nm. Each measurement was performed for three samples in parallel. The percentage of free rifabutin was calculated relative to the initial total content of rifabutin (M_t_/M_∞_ × 100%).

### 2.6. Dissolution Kinetics of HSA-Stabilized Rifabutin Nanoparticles

HSA-stabilized rifabutin nanoparticles were resuspended in MQ-water (type I/ water for injections) at a concentration of 5 mg⸱mL^−1^ for rifabutin and further diluted with MQ-water up to 200 times. Immediately after dilution, the mean size was measured by dynamic light scattering (Zetasizer Nano ZS, Malvern Instruments, UK). Formation of HSA-rifabutin complex solution and disintegration of rifabutin nanoparticles were observed as the disappearance of the peak around 300–400 nm and the appearance of the main peak of 5–7 nm corresponding to the size of an albumin molecule.

### 2.7. Pharmacokinetic Study

All experiments were carried out in compliance with the guidelines of the European Convention for the Protection of Vertebrate Animals, Directives 86/609/EEC, recommendations of the FELASA (Federation of European Laboratory Animal Science Associations) working group (1986, 86/609/EEC, ISSN 03780 6978), and the National standard of the Russian Federation R 53434-2009 “Good Laboratory Practice”. The study was performed on rats and rabbits.

### 2.8. Rats (Pharmacokinetics and Body Distribution)

A total of 120 adult female Wistar rats (150–180 g) were obtained from the animal production unit of N.N. Blokhin Russian Cancer Research Center (Moscow, Russia). The rats were caged in groups of six and maintained on a standard 12-h light-dark cycle. They received standard laboratory food and water ad libitum throughout the study. The rifabutin-loaded PLGA nanoparticles and HSA-stabilized rifabutin nanoparticles formulations were injected via IV into the lateral tail vein of female Wistar rats (150–180 g) at a dose of 6.25 mg/kg, considered the highest therapeutic dose (corresponding to the ~1/8 LD_50_ evaluated in the acute toxicity study) [15]. The suspensions were prepared immediately before the injection by resuspension of the freeze-dried formulations in water for injections at a concentration of 5 mg·mL^−1^ (rifabutin). The animals were sacrificed 5, 15, and 30 min, and 1, 3, 5, 7, and 24 h after administration (*n* = 6 for each time point). The samples of blood, tissues, and organs (liver, kidney, lung, spleen, muscle, and brain) were collected. The organs were separated, minced with eye scissors in Petri dishes placed on ice, and the weighed portions of 500 ± 1 mg were prepared. Then 2.5 mL of distilled water and 250 μL of 0.1 M phosphate buffer (pH = 7.4) were added, and the samples were homogenized using a glass Glas-ColÒ tissue homogenizer (Glas-Col LLC, Terre Haute, IN, USA). For the spleen, the weighed portion was 300 ± 1 mg, and the amount of water and buffer for homogenization was decreased proportionally. Further, an aliquot (600 μL), equivalent to 100 mg of tissue, was taken from the homogenate using a Microman^®^ 1000 pipette (Gilson, Middleton, WI, USA) and quantitatively transferred into vials. An amount of 1.5 mL acetonitrile was added and vortexed for 2 min (Heidolph Reax Top, Schwabach, Germany) then ultrasonicated for 10 min (US-bath Branson B-12). Blood plasma was separated by centrifugation at 2000× *g* rpm for 15 min. To 200 μL of blood plasma, 100 μL of 0.1 M phosphate buffer (pH = 7.4) was added and shaken, then 600 μL of acetonitrile was added, placed on a shaker for 2 min, and transferred to an ultrasonic bath for 10 min.

All samples were then centrifuged at 4000× *g* rpm for 10 min and placed in a refrigerator at +5 °C for 40 min. A total of 170 μL supernatant was taken, and 200 μL of buffer was added (200 mg of Na_2_EDTA salt per 1000 mL of distilled water with the addition of 2 mL of trifluoroacetic acid), shaken, and centrifuged for 15 min at 4000× *g* rpm (“working solution”). The samples were filtered and then injected (50–100 μL) into the HPLC system.

The calibration standards were prepared using rat plasma and organ homogenates as described above. The calibration curves were linear over the range of 0.1–25 μg⸱ mL^−1^ for rat plasma and 0.2–50 μg·g^−1^ for organ tissue. For the brain, the detection limit was 0.5 μg·g^−1^.

The efficacy of the extraction procedures was determined in the model experiment when a known amount of rifabutin was added to the blood and then submitted to the same above-mentioned extraction protocol. The calculated efficacy was 85 ± 4%.

### 2.9. HPLC Analysis

The rifabutin concentrations in the samples were assessed using an Agilent HPLC system (1200 series, Agilent Technologies, Santa Clara, CA, USA) equipped with a diode array detector, a Zorbax SB C18 column (150 × 3 mm, 5 μm), and a Zorbax XDB C8 guard column (2.1 × 12 mm, 5 μm) connected in line according to the back flash scheme. The mobile phase consisted of a mixture of acetonitrile and a buffer solution (200 mg EDTA disodium salt per 1000 mL with the addition of 2 mL of trifluoroacetic acid) in a volume ratio of 65:60. The flow rate of the eluent was 0.4 mL/min, the injected volume was 50–100 µL of the “working solution”; the detection wavelength was 275 nm.

### 2.10. Rabbits (Linearity of the Plasma Pharmacokinetics)

Additionally, the linearity of the plasma pharmacokinetics was studied in female Chinchilla rabbits (2.5 kg) after a single IV injection into an ear vein in 3 doses: 2.76 mg/kg (40.6 mg/m^2^), 5.52 mg/kg, and 11.4 mg/kg (*n* = 5–6). The commercial formulation of rifabutin (RFB-OZON^®^, capsules 150 mg, OZON Ltd., Zhigulevsk, Russia) used as a control was administered orally at a dose of 2.76 mg/kg. The blood samples were collected 5, 15, and 30 min, and 1, 2, 3, 5, 7, and 24 h after administration. The blood plasma was obtained by centrifugation at 2000× *g* rpm for 15 min. To 200 μL of blood plasma, 100 μL of 0.1 M phosphate buffer (pH 7.4) was added. A volume of 600 μL of acetonitrile was added to the sample and it was placed on a shaker for 2 min. Afterward, the sample was transferred to an ultrasonic bath for another 10 min before further processing as described above.

### 2.11. Model Analysis of the Biodistribution

#### 2.11.1. Non-Compartmental Analysis

The pharmacokinetics of the two nanoparticle species, namely, HSA-stabilized rifabutin nanoparticles and rifabutin-loaded PLGA nanoparticles, were analyzed using Monolix2021R1 Suite (Lixoft, Antony, France). Initially, a non-compartmental analysis (NCA) of the plasma pharmacokinetics in rats and rabbits was conducted in PKanalix followed by the calculation of statistically relevant differences using the bioequivalence module. Inequivalence between the two formulation prototypes was estimated by calculating the relative (percentage) difference between the two prototypes including the pharmacokinetic parameters lambda_z, C_max_, AUC_last_ AUC_INF_, Cl_obs_, and V_obs,_ and the parameters were ranked accordingly. Additionally, the pharmacokinetic parameters for different dose levels of the same formulation were compared. To allow a direct comparison, the dose fractions per milliliter were calculated for each dose level.

The relative (percentage) difference from the average of two parameters (e.g., C_max_ of two formulations, dose levels) was calculated as indicated in Equation (2):

(2) $$\mathrm{Relative}\;\mathrm{difference}\;[\%]=\frac{\left|x_{1}-{\;x}_{2}\right|}{0.5\times \left|x_{1}+{\;x}_{2}\right|}\times 100$$

where x_1_ denotes the pharmacokinetic parameter of test formulation or dose level, and x_2_ is the respective reference.

#### 2.11.2. Compartmental Analysis

Compartmental analysis was carried out using Monolix 2021R1 (Lixoft, Antony, Paris, France). The physiologically-based nanocarrier biopharmaceutics (PBNB) model was applied as described previously [11,12,13,14]. The pharmacokinetics in humans and larger animals are often characterized by a delayed t_max_ after the end of the infusion. This is due to the initial vascular distribution phase and has not been observed in smaller species. Hence, we used the modified model design without a vascular transit compartment as described by Kovshova et al. [11]. To estimate the distribution and elimination parameters of the free drug, the pharmacokinetics of HSA-stabilized rifabutin particles were analyzed at the lowest dose strength using Pkanalix (Lixoft, Antony, Paris, France). There is a certain uncertainty related to the underlying assumption that rifabutin occurs predominantly in the free fraction. However, since all formulations are deconvoluted based on the same dataset, this mainly affects the accuracy of the estimated absolute in vivo release and not the relative quantitative comparison between the formulation prototypes. When detecting inequivalence, the compartmental model mainly serves as a background correction of carrier-related elimination and distribution parameters regarding the influence of the free drug.

### 2.12. Statistical Analysis

Statistical data processing was performed using the Microsoft Office 365 software package (Microsoft, Redmont, Birmingham, AL, USA) and Monolix Suite 2021R1 (Lixoft, Antony, Paris, France) including PKanalix for NCA and bioequivalence calculations and Monolix for compartmental analysis. The PBNB model was programmed in Mlxtran.

## 3. Results and Discussion

A variety of nanomaterial-based injectable drug products are under development. To evaluate differences and similarities between formulation prototypes, biopharmaceutical characterization involves physicochemical and in vitro characterization as well as pharmacokinetic analysis. In the current approach, we developed two nanoparticle formulations of rifabutin and evaluated the suitability of NCA and model-based pharmacokinetic analysis to quantify existing differences.

### 3.1. Nanoparticle Characteristics

Initially, two formulation prototypes of rifabutin for intravenous injection were prepared including HSA-stabilized drug nanoparticles and drug-loaded PLGA nanoparticles. All physicochemical characteristics are presented in Table 1. Encapsulation of the antibiotic into PLGA resulted in nanoparticles with a size of approximately 100 nm and a negative surface charge (−25 to −30 mV). Hence, the colloidal dispersion is stabilized by the inhibition of agglomeration through the presence of HSA molecules as well as the repulsion forces of the negatively charged particles. The encapsulation efficiency reached 85–90% at a drug-to-polymer ratio of 1:2.5. Moreover, the PDI of approximately 0.2 indicated a monodisperse distribution.

HSA-stabilized drug nanoparticles were obtained by nanoprecipitation and broadly distributed in size. The zeta potential of −10–15 mV was slightly lower as compared to PLGA nanoparticles. The size and charge are both likely to affect the biodistribution of nanoparticles. Particles with a higher zeta potential often interact with biological surfaces. At the same time, the repulsion forces stabilize the dispersion during storage.

**Figure 1:**
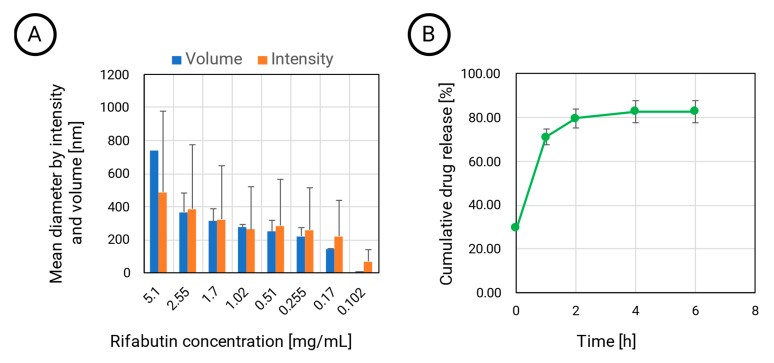
The mean particle diameter of HSA-stabilized rifabutin particles by intensity and volume distribution (**A**). At high dilution, a diameter of approximately 8 nm indicates that rifabutin is converted into the monomolecular form (*n* = 3, Mean ± SD). The cumulative drug release profile of rifabutin-loaded PLGA nanoparticles in 0.15 M PBS (pH 7.4) supplemented with 0.1% ascorbic acid (25-fold dilution, 37 °C, *n* = 3, *p* = 0.95) (**B**).

Interestingly, the particle diameter depends on the dilution. While PLGA provides a stable matrix structure, the HSA-stabilized nanoparticles are the assembled agglomerates that dissolve at lower concentrations. At the lowest tested concentration of approximately 0.1 mg·mL^−1^, the intensity diameter still indicated the presence of the larger assemblies with sizes of more than 70 nm (Figure 1A).

**Figure 2:**
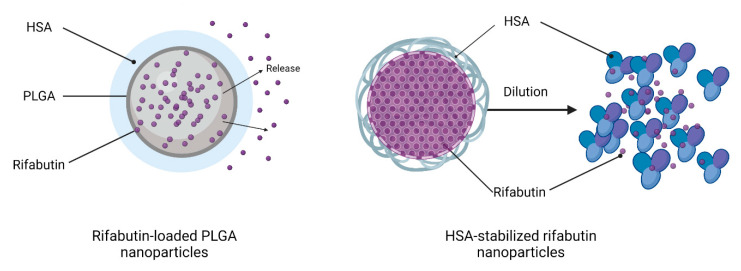
Structure of the two formulation prototypes including rifabutin-loaded PLGA nanoparticles (**left**) and HSA-stabilized rifabutin nanoparticles (**right**). While the PLGA particles provide a stable matrix structure, HSA-stabilized rifabutin nanoparticles disassemble into smaller particles when diluted with water. Created with BioRender.com. https://app.biorender.com/ (accessed on 22 February 2023).

However, when calculating the particle diameter by volume, the size decreased to 8 nm, which indicates that a certain fraction of rifabutin has been solubilized by albumin, which now predominantly occurs in the monomolecular form. An illustration of this disassembly is presented in Figure 2 (right). This certainly affects the biodistribution patterns of these nanoparticles after injection. On the contrary, the size of rifabutin-loaded PLGA nanoparticles was not affected by the dilution and remained within the initial size range (Table 1). The drug release of these nanoparticles was further tested in 0.15 M PBS supplemented with 0.1% ascorbic acid (Figure 1B). Approximately 80% of the drug was released within the first two hours. Ascorbic acid is an antioxidant and inhibits the oxidation of rifabutin, a common process observed for other rifamycin derivatives as well [16,17].

### 3.2. Non-Compartmental Analysis (NCA) of Plasma Pharmacokinetics

As described in the earlier sections, two nanoformulations of rifabutin were prepared. Both delivery systems were suitable for intravenous administration and led to a considerable increase of the rifabutin content in the aqueous phase (6.1 µg⸱mL^−1^ for HSA-stabilized nanoparticles and 3.6 µg⸱mL^−1^ for PLGA nanoparticles) as compared to the macrocrystalline drug substance (0.19 µg⸱mL^−1^). However, differences in their physicochemical properties resulted in different release profiles. The release kinetics and particle size of nanoparticles are likely to affect biodistribution. Accordingly, we assumed biopharmaceutical inequivalence. Hence, we conducted a biodistribution study in rats to compare in vivo performances. NCA was used to evaluate the capability of common pharmacokinetic parameters to quantify biopharmaceutical inequivalence between the two formulation prototypes.

#### 3.2.1. Comparison of Biodistribution

**Figure 3:**
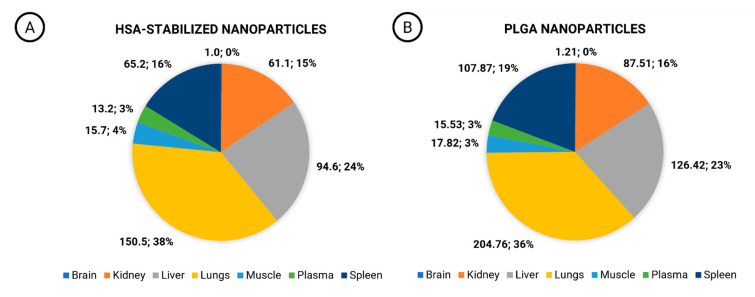
Comparison of the percentage exposure of HSA-stabilized rifabutin nanoparticles (**A**) and PLGA nanoparticles (**B**) based on AUC_last_ (h⸱µg⸱mL^−1^), *n* = 6. The study served as confirmation that differences in plasma pharmacokinetics are a reflection of differences in their biodistribution and, due to their material attributes, both formulations are inequivalent regarding their in vivo performances.

Biodistribution studies are almost exclusively carried out during preclinical research and often do not qualify for the evaluation of inequivalence during clinical trials. In the current investigation, inequivalence was revealed by quantification of the drug exposure detected in the brain, kidneys, liver, lungs, muscle tissue, and spleen of rats (Figure 3A,B). Both nanoformulations exhibited comparatively low plasma AUC and extensive tissue distribution that was observed in earlier studies for conventional rifabutin formulations as well [2]. Although the relative exposure levels (%) of rifabutin in these organs appeared to be similar, the organ AUC was significantly different. These significant differences indicate the different biodistribution profiles of both formulations. Although there was a minor shift in the percentage distribution only (38% of the exposure for HSA-stabilized nanoparticles, and 36% for PLGA nanoparticles), drug concentrations in the lungs increased significantly for PLGA nanoparticles (Figure 3B: 204.75 h⸱µg⸱mL^−1^) as compared to HSA-stabilized drug nanoparticles (Figure 3A). Additionally, the exposure level in the spleen increased from 65.2 h⸱µg⸱mL^−1^ for HSA-stabilized nanoparticles (Figure 3A) to 107.87 h⸱µg⸱mL^−1^ for PLGA nanoparticles (Figure 3B). A similar tendency was observed in the liver exposure (94.6 h⸱µg⸱mL^−1^ and 126.42 h⸱µg⸱mL^−1^, respectively).

The lungs are the target site of rifabutin therapy and therefore play a major role in the estimation of efficacy levels. Additionally, the lungs represent an important organ that is known to accumulate nanoparticles due to the fine capillaries that filter colloidal carriers from blood circulation. An accumulation in the spleen and liver is also an important characteristic of rifabutin [18], and it is further enhanced by the cellular uptake of the nanoparticles by the macrophages which may indicate differences in particle elimination from the blood plasma.

#### 3.2.2. Comparison of Formulations

Most guidelines use AUC and c_max_ for bioequivalence assessment. Considering the potential limitations of the current framework for the evaluation of nanomedicines, additional parameters, such as lambda_z (h^−1^), Cl_obs_ (mL⋅h^−1^), V_obs_ (mL), were included. Lambda_z (h^−1^) is an elimination rate constant calculated from the terminal phase of the pharmacokinetic profile. Cl_obs_ (mL⋅h^−1^) is the observed drug clearance, which is the rate at which the drug is eliminated from the body relative to its concentration in the bloodstream. They both represent elimination parameters. V_obs_ (mL) further represents an important distribution parameter that often changes with particle size. After their injection, nanomedicines undergo a particle-size-dependent distribution and elimination process that is likely to affect all three parameters [11,12,13,14].

The outcome of the NCA is presented in Table 2. Because of the different dose strengths and physiology of the animals, we compared the relative difference between the formulations and dose strengths obtained from the same species without conducting an interspecies comparison. In rats, the volume of distribution (V_obs_) and lambda_z, the slope of the terminal elimination phase, detected differences between the two prototypes most sensitively (ranks 1/5 and 2/5). Lambda_z is one of the above-mentioned elimination parameters. Unfortunately, a similar influence on the calculated clearance could not be observed (rank 5/5). C_max_ was one of the most sensitive parameters in rats (rank 3/5). It is affected by the absorption, elimination, and volume of the distribution of drugs; hence, it was introduced as one of two bioequivalence criteria in most regulatory frameworks.

In rabbits, the sensitivity of the elimination parameter Lambda_z was most insensitive to the difference between these two formulation prototypes while C_max_ was in the first rank. AUC_INF_ and Cl_obs_ shared the second rank. Because of the obvious difference in the material properties of PLGA and HSA-stabilized rifabutin nanoparticles, a significant percentage difference between the pharmacokinetic parameters was expected for both experiments. The relative difference between the C_max_ values of the two formulations was 30.5% in rats and 60.7% in rabbits. The AUC differed by 13.8% and 47.7%, respectively.

Interestingly, the rank order of AUC_INF_ and AUC_last_ was not consistent in rats and rabbits. AUC_last_ refers to the AUC from the time of dosing to the last observed concentration, which is typically measured during the elimination phase of the drug. AUC_INF_, on the other hand, refers to the AUC from the time of dosing to infinity, which is extrapolated from the concentration–time curve.

To detect the inequivalence of almost similar formulation prototypes (e.g., generics), a calculation of the partial AUC has been suggested. This more selective comparison of a certain fraction of the pharmacokinetic profile increases the sensitivity for the detection of absolute differences. However, since AUC_last_ represents a partial AUC_INF_, a change in the rank order between two species indicates that even partial AUC calculation could not be generally applied to these nanomedicines.

#### 3.2.3. Comparison of Dose Levels

**Figure 4:**
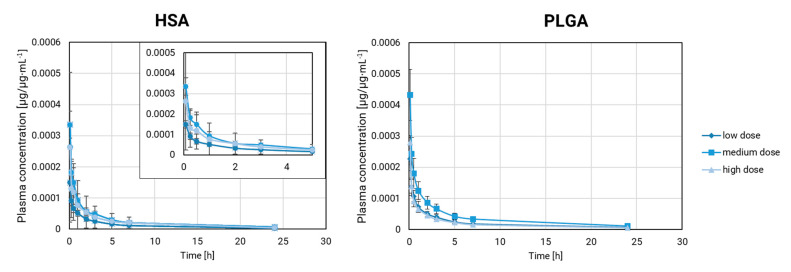
Pharmacokinetics in rabbits: HSA-stabilized rifabutin particles (*n* = 5, Mean ± SD) and rifabutin-loaded PLGA nanoparticles (*n* = 6, Mean ± SD). The dose fraction µg/µg per milliliter is presented to enable a direct comparison of the three formulations at different dose levels. The lowest dose strength of HSA-stabilized rifabutin particles is likely corresponding to the behavior of protein-bound monomolecular rifabutin after injection.

Our comparison of the plasma pharmacokinetics at multiple dose levels in rabbits involved rifabutin-loaded PLGA nanoparticles and HSA-stabilized rifabutin nanoparticles. Drug exposure has a strong impact on metabolism and distribution but also changes the absolute drug concentrations. Accordingly, we carried out our comparison based on the dose fraction per milliliter (µg/µg∙mL^−1^). The resulting fraction-scaled pharmacokinetic profiles are presented in Figure 4.

PLGA nanoparticles remain stable over a wide dose range. Differences in the pharmacokinetic parameters could arise from the different exposure levels with rifabutin as well as the interindividual differences between animals. The dose scaling affects multiple parameters at once and makes it more difficult to identify formulation effects. Firstly, the fraction-scaled medium and lowest dose strength of PLGA nanoparticles were compared. V_obs_ (rank 1) and C_max_ (rank 2) ranked highest in this comparison. All parameters except for Lambda_z fell into a similar range (59–74%, Table 3).

A comparison of the highest and the lowest dose strength of rifabutin-loaded PLGA nanoparticles (Table 3) led to a different outcome. Lambda_z and C_max_ shared the first rank followed by the other parameters. The percentage difference ranged from 28 to 9.3%. By increasing the gap between the two dose strengths, it has become more difficult to detect formulation-related differences. This evaluation makes the weakness of the conventional pharmacokinetic parameters more apparent. Every difference in the population, metabolism, or exposure level is likely to affect the sensitivity of inequivalence testing.

Considering PLGA nanoparticles at multiple dose levels as a pharmaceutically equivalent negative control, we performed a similar comparison for HSA-stabilized rifabutin nanoparticles. For this formulation prototype, the dilution at various dose strengths is more likely to induce formulation-related differences.

The NCA of HSA-stabilized rifabutin nanoparticles (Table 4) indicated a pronounced difference in the C_max_ for both dose strength comparisons (rank 1). However, as observed for PLGA nanoparticles, the rank order changes for each comparison, and differences between various parameters are not pronounced enough to identify a significant formulation-related difference.

Still, our findings provide a certain justification for the current bioequivalence framework. C_max_ and AUC consistently detect differences between inequivalent formulations, but their sensitivity is very limited. The inequivalence between the nanoparticle formulation prototypes under investigation is most likely to affect C_max_. This aligns with current theories that encapsulation and burst release quantitatively play an important role in this kind of product.

As a next step, we explored mechanistic compartmental modeling. Here, the deconvolution provides more resolved pharmacokinetic parameters which can be corrected for certain influences (e.g., drug elimination). On the one hand, this often provides a more sensitive detection through deconvolution and background correction. However, the assumptions underlying this analysis must similarly apply to all formulations under investigation. Hence, there is a certain risk of additional systematical errors that may affect the outcome.

### 3.3. Mechanistic Compartmental Modeling of the Plasma Pharmacokinetics in Rabbits

**Figure 5:**
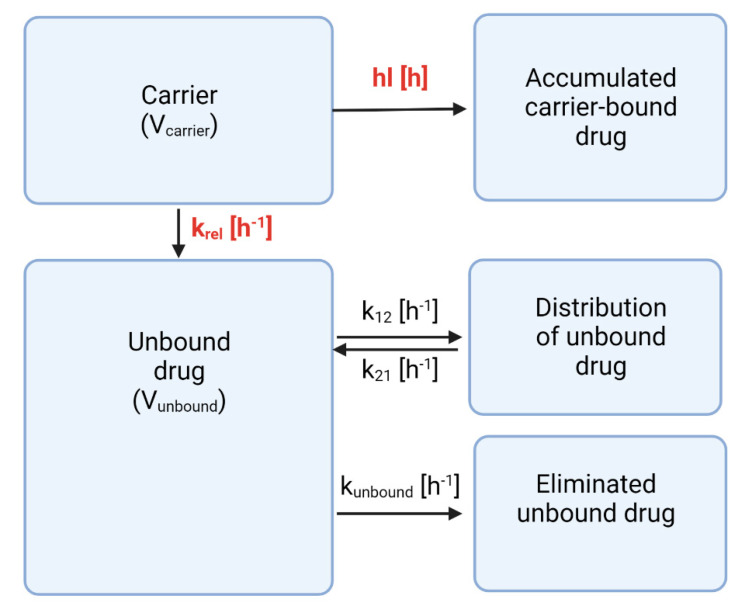
Illustration of the PBNB model that was used for the compartmental analysis (hl = Carrier half-life; krel = Release rate of doxorubicin in vivo; V_carrier_ = Volume of distribution of the carrier; V_unbound_ = Volume of distribution of doxorubicin; k12, k21 = Circulation and recirculation rates of doxorubicin into the periphery; k_unbound_ = Elimination rate of doxorubicin).

For the current investigation, our compartmental analysis was based on the PBNB model (Figure 5) [11,12,13,14]. To facilitate this deconvolution, pharmacokinetic parameters of the unbound drug are required [11,12,13,14]. Rifabutin is a poorly soluble molecule that does not allow the administration of the same dose without further solubilization.

However, the synthesis of rifabutin drug nanoparticles was based on the *nab*-technology that has been used to manufacture the commercial drug product Abraxane^TM^ previously [19,20,21,22]. Upon dilution, a significant fraction of HSA-stabilized colloid disassembles into drug-protein complexes. As a consequence, at the lowest dose strength (and highest dilution), these particles are likely to exhibit distribution and elimination parameters very similar to the protein-bound drug. Considering a plasma volume of 99.1 mL for a 2.5 kg rabbit [23], the lowest dose strength corresponds to an initial rifabutin concentration after injection of 69.6 µg∙mL^−1^. This is even below the lowest rifabutin concentration tested in our dilution experiment. Moreover, the drug exhibits a plasma protein binding of 85%, which further supports our assumption [24]. Consequently, the pharmacokinetic parameters of the free drug were obtained by fitting the pharmacokinetics of HSA-stabilized rifabutin nanoparticles at the lowest dose strength (Table 5).

Noteworthy, the presence of partially particle-bound rifabutin represents an error source in our deconvolution. However, this error would mainly affect the prediction of the real in vivo release of rifabutin and not a relative comparison between formulation prototypes.

Estimation of the parameters was achieved by fitting the mean plasma pharmacokinetics with a two-compartment model. They were used as the initial estimates (Table 5) of the compartmental analysis. The volume of distribution of the carrier was estimated from the plasma volume of rabbits [23]. However, while the transport rates and their standard deviations were fixed to the mean values and standard deviations provided in Table 5, likelihood estimations were conducted for the volumes of distribution (V_carrier_ and V_unbound_).

The PBNB model was applied to the same dose-normalized datasets of HSA-stabilized rifabutin nanoparticles and rifabutin-loaded PLGA nanoparticles described previously (Section 3.2). The analysis mainly identifies two formulation-dependent parameters—the release rate (k_rel_) and the carrier half-life (hl)—while other parameters can be associated to the drug molecule (Supplementary Materials, Table S1).

**Figure 6:**
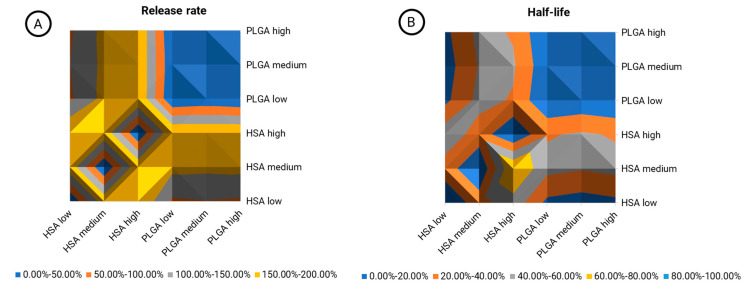
Inequivalence contour map indicating the relative (percentage) difference between formulation prototypes (PLGA vs. HSA) and their respective dose strengths (low, medium, high) in different colors. The comparison was conducted for the formulation-related parameters obtained from the PBNB model including the in vivo release rate (**A**) and the carrier half-life (**B**).

The inherent material properties of PLGA and HSA-stabilized rifabutin nanoparticles are most likely to lead to differences in their pharmacokinetics. Not only do they affect their chemistry but also their size, morphology, and other physicochemical characteristics. An inequivalence contour map was designed to show the relative (percentage) difference between formulations and dose levels (Figure 6). The release rate (Figure 6A) and half-life (Figure 6B) are both illustrated separately.

The blue area in the top right quarter of both graphs (Figure 6A,B) shows a comparison of PLGA nanoparticles at three different dose levels. Expectedly, the average variation in this area was 3.87% in the release rate and 2.02% in the carrier half-life, as indicated by the color (0–50.00%). Hence, the model parameters did not respond to dose-related changes that do not affect the carrier properties. As we learned from the physicochemical and in vitro characterization of HSA-stabilized rifabutin nanoparticles, for these particles, the dose strength likely affects the size distribution and release behavior of the formulation, too. Hence, a similar comparison between the dose strengths of HSA-stabilized nanoparticles led to much higher average differences of 128.30% (release rate,/Figure 6A, bottom left quarter) and 31.11% (half-life/Figure 6B, bottom left quarter). The average differences between PLGA and HSA-stabilized nanoparticles were 152.46% (release rate/Figure 6A, top left and bottom right quarter) and 32.50% (half-life/Figure 6B, top left and bottom right quarter), respectively. Overall, it is not surprising that the carrier half-life was less affected. The disassembly of HSA nanoparticles into the albumin-bound molecules will potentially occur much faster in vivo due to the distribution and dilution of the carrier. As compared to the NCA, inequivalence was detected much more sensitively. Moreover, the reasons for biopharmaceutical inequivalence were reflected by the pharmacokinetic parameters indicating improved robustness against population-dependent influences.

## 4. Conclusions

Two intravenous formulation prototypes of rifabutin with inherently different drug release patterns have been developed. Rifabutin-loaded PLGA nanoparticles provided a stable matrix structure with a well-defined monodisperse particle size distribution that retained approximately 50% of the encapsulated drug for at least 1 h after injection. On the contrary, HSA-stabilized rifabutin nanoparticles undergo immediate disassembly upon dilution, which reduces their particle size and affects in vivo performance at different dose strengths significantly. We systematically evaluated the sensitivity of NCA and mechanistic compartmental analysis using the PBNB model in detecting biopharmaceutical inequivalence by comparing these two inequivalent formulations and their dose fraction-scaled pharmacokinetic profiles. 

NCA confirmed the inequivalence between the formulations but could not differentiate between formulation- and dose-related influences. Remarkably, despite having identical formulation parameters, the selected dose strengths of PLGA nanoparticles (low, medium, high) resulted in relative differences of 66.7% (low vs. medium) and 19.6% (low vs. high), indicating limited robustness of this technique in resolving formulation-related inequivalence. Partial AUC calculation, which is often recommended to enhance the sensitivity of NCA, was not successful for the formulations under investigation, as indicated by the rank order of AUC_inf_ and AUC_last_ (which can be considered as a partial AUC of AUC_inf_). In contrast, the PBNB model resolved formulation-related differences using parameters such as in vivo release rate and carrier half-life. The average difference of 152.46% between PLGA and HSA-stabilized nanoparticles (across all dose strengths) was identified, while the comparison of different dose strengths of PLGA nanoparticles, on average, showed a minimal 3.87% difference, suggesting a reduced influence of drug-related parameters on the analysis outcome. This study demonstrates the superior sensitivity and high robustness of mechanistic compartmental analysis in the evaluation of nano-medicines and highlights a new approach for the analysis of nanosimilars.

## Figures and Tables

**Table 1:** Physicochemical parameters of rifabutin formulation prototypes.

Formulation	Mean Particle Size [nm]	Polydispersity Index	Zeta Potential [mV]	Encapsulation Efficiency [%]	Rifabutin Content [mg⸱mL^−1^]
HSA-stabilized rifabutin nanoparticles	337 ± 47	0.329 ± 0.096	−10–15	88–92	6.1 ± 0.4
Rifabutin-loaded PLGA nanoparticles	100 ± 13	0.199 ± 0.020	−27–29	85–90	3.6 ± 0.2

**Table 2:** NCA evaluation of the pharmacokinetics of HSA-stabilized rifabutin nanoparticles and rifabutin-loaded PLGA nanoparticles in rats (*n* = 6 for the HSA and PLGA groups) and rabbits (*n* = 6 for the HSA group and *n* = 5 for the PLGA group) at the lowest dose strength using the PKanalix bioequivalence module. The parameters have been ranked by the relative difference between the two formulation prototypes.

Comparison of Rifabutin-Loaded PLGA Nanoparticles (PLGA) and HSA-Stabilized Rifabutin Nanoparticles (HSA) in Rats	Adjusted Mean (HSA)	Adjusted Mean (PLGA)	Relative (Percentage) Difference [%]	Rank
V_obs_ (mL)	696.84	1084.65	43.5%	1
Lambda_z (h^−1^)	0.079	0.055	35.8%	2
C_max_ (µg⋅mL^−1^)	4.69	3.45	30.5%	3
AUC_last_ (h µg⋅mL^−1^)	15.08	13.13	13.8%	4
Cl_obs_ (mL⋅h^−1^)	54.93	60.12	9.0%	5
AUC_INF_ (h µg⋅mL^−1^)	18.77	17.15	9.0%	5
Comparison of the Highest Dose of Rifabutin-Loaded PLGA Nanoparticles (PLGA) and HSA-Stabilized Rifabutin Nanoparticles (HSA) in Rabbits	Adjusted Mean (HSA)	Adjusted Mean (PLGA)	Relative (Percentage) Difference [%]	Rank
C_max_ (µg⋅mL^−1^)	7.97	4.26	60.7%	1
AUC_INF_ (h µg⋅mL^−1^)	16.81	10.34	47.7%	2
Cl_obs_ (mL⋅h^−1^)	1695.48	2755.92	47.6%	3
AUC_last_ (h µg⋅mL^−1^)	14.44	9.42	42.1%	4
V_obs_ (mL)	23,272	31,878.3	31.2%	5
Lambda_z (h^−1^)	0.073	0.086	16.4%	6

**Table 3:** NCA evaluation of the pharmacokinetics of rifabutin-loaded PLGA nanoparticles in rabbits (*n* = 5) using the PKanalix bioequivalence module. The different dose strengths were compared based on the dose fractions per milliliter and all parameters were calculated accordingly.

Comparison of Medium (M) and Lowest (L) Dose of Rifabutin-Loaded PLGA Nanoparticles	Adjusted Mean (M)	Adjusted Mean (L)	Relative (Percentage) Difference [%]	Rank
V_obs_ (mL)	11,536.7	24,116.3	70.6%	1
C_max_ (µg/µg⋅mL^−1^)	0.00046	0.00023	66.7%	2
AUC_last_ (h⋅µg/µg⋅mL^−1^)	0.0011	0.00057	63.5%	3
AUC_INF_ (h⋅µg/µg⋅mL^−1^)	0.0013	0.00071	58.7%	4
Cl_obs_ (mL⋅h^−1^)	773.3	1401.63	57.8%	5
Lambda_z (h^−1^)	0.067	0.058	14.4%	6
Comparison of Highest (H) and Lowest (L) Dose of Rifabutin-Loaded PLGA Nanoparticles	Adjusted Mean (H)	Adjusted Mean (L)	Relative (Percentage) Difference [%]	Rank
Lambda_z (h^−1^)	1710.77	1289.35	22.9%	1
AUC_INF_ (h⋅µg/µg⋅mL^−1^)	0.0005	0.00063	20.2%	2
Cl_obs_ (mL⋅h^−1^)	0.073	0.06	19.9%	3
C_max_ (µg/µg⋅mL^−1^)	0.00058	0.00078	19.6%	4
AUC_last_ (h⋅µg/µg⋅mL^−1^)	0.00028	0.00025	13.1%	5
V_obs_ (mL)	23,483.1	21,397.9	2.7%	6

**Table 4:** NCA evaluation of the pharmacokinetics of HSA-stabilized rifabutin nanoparticles in rabbits (*n* = 5) using the PKanalix bioequivalence module. The different dose strengths were compared based on the dose fractions per milliliter.

Comparison of the Lowest (L) and Medium (M) Dose of HSA-Stabilized Rifabutin Nanoparticles	Adjusted Mean (M)	Adjusted Mean (L)	Relative (Percentage) Difference [%]	Rank
C_max_ (µg/µg⋅mL^−1^)	0.00033	0.00014	80.9%	1
AUC_INF_ (h⋅µg/µg⋅mL^−1^)	0.00076	0.0004	62.1%	2
Cl_obs_ (mL⋅h^−1^)	1322	2492.02	61.4%	3
AUC_last_ (h⋅µg/µg⋅mL^−1^)	0.00067	0.00036	60.2%	4
V_obs_ (mL)	17,142.5	30,684.7	56.6%	5
Lambda_z (h^−1^)	0.077	0.081	5.1%	6
Comparison of the Lowest and Highest Dose of HSA-Stabilized Rifabutin Nanoparticles	Adjusted Mean (H)	Adjusted Mean (L)	Relative (Percentage) Difference [%]	Rank
C_max_ (µg/µg⋅mL^−1^)	0.0002	0.00014	35.3%	1
V_obs_ (mL)	23,982.2	30,684.7	24.5%	2
AUC_last_ (h⋅µg/µg⋅mL^−1^)	0.00044	0.00036	20.0%	3
Cl_obs_ (mL⋅h^−1^)	2074.21	2492.02	18.3%	4
AUC_INF_ (h⋅µg/µg⋅mL^−1^)	0.00048	0.0004	18.2%	5
Lambda_z (h^−1^)	0.086	0.081	6.0%	6

**Table 5:** Initial estimates for the compartmental analysis of the plasma pharmacokinetics of HSA-stabilized rifabutin nanoparticles and rifabutin-loaded PLGA nanoparticles. The analysis was conducted with fixed parameters and SD settings as indicated below.

Parameter	Mean	SD	Source
k_unbound_ [h^−1^]	0.37	0.02	Analysis of rifabutin pharmacokinetics
k_12_ [h^−1^]	0.91	0.03	Analysis of rifabutin pharmacokinetics
k_21_ [h^−1^]	0.35	0.01	Analysis of rifabutin pharmacokinetics

## Data Availability

No new data were created or analyzed in this study. Data sharing is not applicable to this article.

## Supplementary Materials

The following supporting information can be downloaded at: https://www.mdpi.com/article/10.3390/pharmaceutics15041258/s1, Table S1: Release rate and carrier half-life identified by the PBNB model for the various formulations. The relative (percentage difference) was calculated to create the contour map.
