# Supplementary material for: Comparison of Compartmental and Non-Compartmental Analysis to Detect Biopharmaceutical Similarity of Intravenous Nanomaterial-Based Rifabutin Formulations
Source: Pharmaceutics. 2023 Apr 17;15(4):1258. doi: 10.3390/pharmaceutics15041258 (PMC10145013; doi:10.3390/pharmaceutics15041258)
Supplement: Supplementary file 1 [file pharmaceutics-15-01258-s001.zip › pharmaceutics-2331259-supplementary.pdf]

Supplementary material to the article:

Comparison of Compartmental and Non-Compartmental Analysis to Detect Biopharmaceutical Similarity of Intravenous Nanomaterial-based Rifabutin Formulations

Nadezhda Osipova, Andrey Budko, Olga Maksimenko, Elena Shipulo, Ludmila Vanchugova, Wenqian Chen, Svetlana Gelperina, Matthias G. Wacker
Correspondence should be directed to svetlana.gelperina@gmail.com
and matthias.g.wacker@nus.edu.sg

Table S1. Release rate and carrier half-life identified by the PBNB model. The values were used to calculate the relative (percentage) difference in the contour map.

**Formulation**	**k_{rel} [h^{-1}]**	**SD**	**Carrier half-life [h]**	**SD**
**HSA low**	460.1	10.2	0.00055	0.00000
**HSA medium**	16.3	0.0	0.00040	0.00000
**PLGA high**	0.4	0.0	0.00094	0.00000
**PLGA low**	170.0	17.0	0.00064	0.00008
**PLGA medium**	155.9	14.3	0.00061	0.00008
**PLGA high**	170.1	34.8	0.00064	0.00007
